# Health damage assessment of commuters and staff in the metro system based on field monitoring—A case study of Nanjing

**DOI:** 10.3389/fpubh.2023.1305829

**Published:** 2024-01-11

**Authors:** Shu Su, Shuhao Li, Yujie Ding, Peng Mao, Dan Chong

**Affiliations:** ^1^Department of Construction and Real Estate, School of Civil Engineering, Southeast University, Nanjing, China; ^2^Department of Engineering Management, School of Civil Engineering, Nanjing Forestry University, Nanjing, China; ^3^Department of Management Science and Engineering, School of Management, Shanghai University, Shanghai, China

**Keywords:** metro station, health damage assessment, field monitoring, air pollution, case analysis

## Abstract

**Introduction:**

The metro has emerged as a major mode of transportation. A significant number of commuters and staff in the metro system are exposed to air pollutants because of its shielded environment, and substantial health damage requires quantitative assessment. Previous studies have focused on comparing the health impacts among different transportation modes, overlooking the specific population characteristics and pollutant distribution in metro systems.

**Methods:**

To make improvements, this study implements field monitoring of the metro's air environment utilizing specialized instruments and develops a health damage assessment model. The model quantifies health damage of two main groups (commuters and staff) in metro systems at three different areas (station halls, platforms, and metro cabins) due to particulate matter 10 and benzene series pollution.

**Conclusion:**

A case study of Nanjing Metro Line 3 was conducted to demonstrate the applicability of the model. Health damage at different metro stations was analyzed, and the health damage of commuters and staff was assessed and compared. This study contributes to enhancing research on health damage in the metro systems by providing a reference for mitigation measures and guiding health subsidy policies.

## 1 Introduction

Traffic in China is becoming more and more congested due to accelerated urbanization (1). The metro system has played an increasingly important role for its advantages such as high-speed, punctuality, and environmental protection (2, 3). By the end of 2021, the total length of China's urban metro lines had reached 7,209 kilometers, and the cumulative passengers reached 23.69 billion (4). As an underground construction, the metro system has the characteristics of relatively closed, inadequate natural ventilation, and dense population. Wheel frictions and coating materials produce large amounts of air pollutants, yet it is typically difficult to discharge them outdoors. As a result, a large number of commuters and metro staffs may be exposed to pollutants, which might cause disease symptoms (5). This contradicts the “*Healthy China 2030*” initiative.

Among the various air pollutants in metro systems, the concentrations (6) and resulting hazards (7–9) of particulate matter 10 (PM10) and Benzene series (BTEX) are considerably high. They are recognized as two major pollutants. PM10 encompasses particles with a size of 10 μm or less, which can arouse health damage after entering the human respiratory system (10–12). Besides, PM10 particles, being larger and characterized by stronger sedimentation, are more prone to accumulate in underground metro environments compared to PM2.5. Therefore, PM10 is one of the main pollutants worth the whistle in metro systems (11, 12). In a metro system, PM10 is mainly generated by friction between metal structures (brake pads, wheels, and others), as well as the operation of air conditioning systems. Particles collected in metro systems contain more metal elements, such as iron and zinc, than those in other air environments; thus, exposure to these particles can be more toxic (13). Exposure to PM10 in metro systems increases the risk of many human diseases, including chronic obstructive pulmonary disease (COPD), cerebrovascular disease (CVD), and acute respiratory infection (14). BTEX mainly originates from insulation and decorative materials (coatings and sprays) inside metro systems. Excessive concentrations of BTEX can cause bodily reactions such as chest tightness, dizziness, headaches, and respiratory irritation (15, 16), which makes it more noteworthy than other pollutants in metro environments.

Many scholars have conducted horizontal comparative analyses of the health issues in metro systems. However, most studies have only analyzed the exposure dose of pollutants (17, 18), and the resulting health damage has not been thoroughly quantified. On the one hand, previous studies primarily focus on the health impacts faced by metro commuters (19). It is worth noting that metro staff, including security inspectors, ticket sellers, and platform attendants, work in a confined metro environment for extended periods each day (~12 h). Metro companies and governments may also need to provide health subsidies to them. This group worths further investigation. On the other hand, field monitoring plays a crucial role in understanding the specific pollutant levels and population exposure in the different areas of metro systems. Existing studies have mainly focused on pollutants on metro platforms (20), while limited attention have been paid to the emissions in station halls and metro cabins. In fact, station halls and metro cabins are typical gathering places for staff and commuters, who may endure prolonged exposure to high pollution concentrations. Therefore, there is currently a lack of research on the systematic evaluation and comparison of health damage in platforms, train cabins, and station halls in the metro system. At the same time, there are gaps in the comprehensive health damage assessment for staff and commuters in the metro system.

The objectives of this study are listed as follows. It hopes to provide a reference for effective measures of damage mitigation in metro systems and helps in occupational health subsidy policymaking.

1) Establishing a health damage assessment model based on field monitoring of pollutants;
2) Applying the proposed assessment model in practice to quantify potential health damage to two groups (commuters and staff) in the metro system;
3) The pollution monitoring and health damage quantification will be conducted and compared at three main sites (station hall, platform, and metro cabin).

The remainder of this study is organized as follows: Section 2 summarizes the current research status and limitations in this field. Section 3 introduces the research method, including field monitoring scheme design and pollutant damage assessment. Section 4 applies the model in practice and analyzes the related results. Section 5 presents the discussion, and Section 6 concludes the study.

## 2 Literature review

### 2.1 Health damage research in metro system

Health issues in metro systems have attracted significant attention with the rapid development worldwide. Some studies have compared the health damage in metro systems with that in other travel modes. Liu et al. (21) analyzed the health hazards of PM in various transportation systems (shared bikes, buses, cars, and metros) in Guangzhou, China, where the PM10 concentration reached 61 μg/m^3^. Tan et al. (22) compared the pollution exposure levels of metros, buses, taxis, and walking in Singapore. However, the studies merely focused on the pollutant concentration level.

Some in-depth studies have focused on metro systems, and the health damage to commuters have been cared more (23). Roy et al. (24) monitored the PM concentration in Pune metro systems (93.7 μg/m^3^), and then the risk of damage from inhalation cancer that commuters may experience at metro stations was evaluated. Shiohara et al. (25) quantified carcinogenic damage caused by volatile chemicals in metro commuters in Mexico. He et al. (26) analyzed the pollution exposure of metro commuters on metro platforms with different protective facilities in Beijing, China. However, very few studies have focused on health damage to staff. Grass et al. (27) analyzed the health damage to maintenance staff in metro systems and found that they were injured by PM10. Owing to prolonged exposure in metro systems, staff are personally damaged far more than commuters and thus need to be concerned.

### 2.2 Field monitoring

As far as the data acquisition method for metro pollutant concentration, field monitoring is a good one and can provide reliable and accurate data. Field monitoring is based on actual observations, and conducting evaluation with actual monitoring data is a research trend (28–30). Usually, a metro system is large and the pollutant levels at different locations (station halls, platforms, and metro cabins) are quite different owing to various different activities. Unfortunately, existing field monitoring have been mainly conducted at specific locations, causing the limitation of reliability of monitoring data. Colombi et al. (31) monitored pollutant concentrations on metro platforms in Milan. The maximum PM10 concentration on the platform of Milan metro station is 283 μg/m^3^, which is much higher than the atmospheric PM10 concentration. Sahin et al. (32) measured PM10 concentration of six stations in Istanbul and the peak concentration of PM10 reached 338.5 μg/m^3^. However, there is a lack of attention to the cabin and station hall. Xu et al. (33) and Zheng et al. (34) analyzed the health damage caused by air pollutants in metro cabins in Shanghai and Xi'an, respectively. Few studies have compared pollutant concentrations in different areas of metro systems (35).

### 2.3 Health damage assessment methods

Human health damage assessment quantifies the possibility of adverse effects caused by chemicals in contaminated environmental media on the health of exposed persons (36). Several methods are commonly used to conduct health damage assessment. One method assesses health damage by monitoring heart rate and estimating ventilation rate. Borghi et al. (37) used this method to calculate the pollutant exposure doses of commuters in the metro and bus systems in Milan. Vale et al. (38) estimated the exposure dose levels of PM10 while walking outdoors in central Lisbon. In addition, the National Institute of Public Health and Environment developed a lung deposition calculation method, which focuses on the damage caused by PM and has been widely used. Roy et al. (11) adopted this method to quantify the health damage in Korean metro systems.

To date, the most widely used human health damage assessment method is the one proposed by the US Environmental Protection Agency (EPA) (39). It consists of four stages: hazard identification, dose-response assessment, exposure assessment, and risk characterization. This method is widely acknowledged for its usability and reliability (40). Additionally, the EPA has established a comprehensive database containing population exposure parameters and pollutant characteristics. This method has been widely used to quantify various health risks, including those caused by molten metals in residents' drinking water (41), carbon dioxide and PM10 in Portuguese primary schools (42), and volatile pollutants in building decoration materials (43).

## 3 Methodology

**Figure 1:**
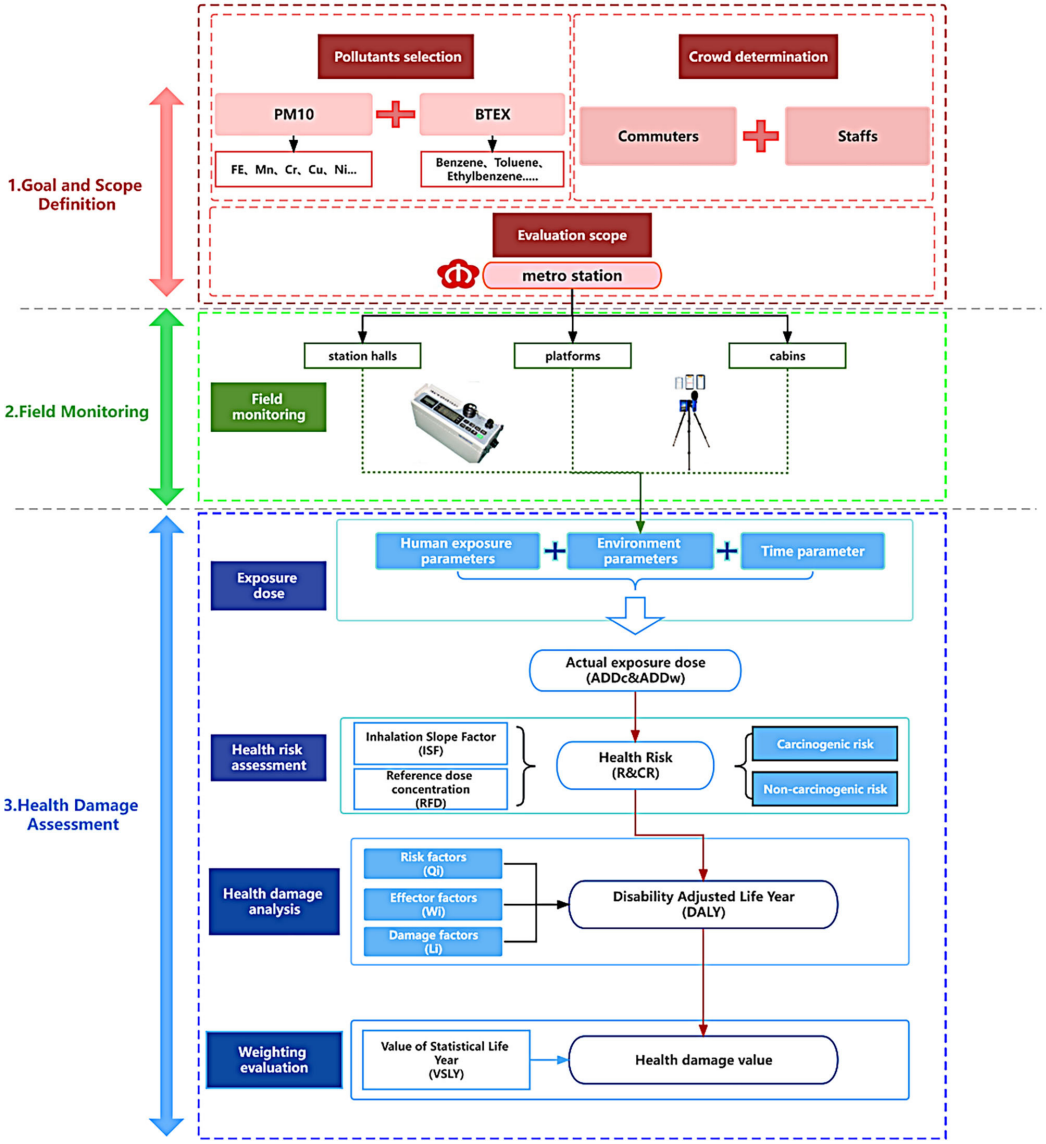
The health damage assessment model for the metro system.

This study aimed to conduct a quantitative assessment of the health impact on commuters and staff members in different areas (including station halls, platforms, and metro cabins) of the metro system. A health damage assessment model was developed that consisted of three modules: goal and scope definition, field monitoring, and health damage assessment. The first module determined the pollutant types, target populations, and monitoring sites. The second module obtained the concentration data of air pollutants in the metro via on-site monitoring. The third module quantified health damage through exposure dose calculations, risk assessments, damage analyses, and weighting evaluation. The research model is depicted in Figure 1.

### 3.1 Goal and scope definition

PM10 and BTEX are the primary air pollutants in urban metro systems, and this study focused on these two types of pollutants. The major causes of health damage include non-carcinogenic diseases (mortality, chronic obstructive pulmonary disease, cardiovascular disease, cerebrovascular disease, and acute respiratory infections) (14, 16) and carcinogenic diseases (mainly leukemia) (44).

The monitored metro stations in this study are located in typical city center areas and suburban regions to ensure the representativeness and applicability of the monitoring results to a broader urban context. These stations are situated underground and share the common characteristics of being enclosed with poor ventilation systems. Field monitoring was conducted to collect pollutant concentration levels, and three typical monitoring sites were selected (station halls, platforms, and metro cabins). The station hall is the area connecting to the entrance/exit, which links the metro cabins and the tunnel, and the metro cabins have the characteristics of a closed space and fast movement. The concentrations of pollutants vary in station halls, platforms, and metro cabins owing to differences in spatial size, population flows, pollutant sources, and other environmental factors (45). Therefore, the hall, platform, and cabin were chosen as monitoring points for assessing the air quality in the microenvironments.

Commuters and staff are the main users of the metro system, and were identified as the assessed population. Commuters represent a diverse group with a wide age distribution and large number of people, whereas metro staff represent a group with long-term exposure to air pollutants, resulting in significant health risks. Staff members were classified into categories according to their workplace, as shown in Table 1.

**Table 1:** Related metro staff at different metro areas.

**Area**	**Staff**
Station hall	Station attendant, watchman, and safety officer
Platform	Driver and driving attendant
Train cabin	Security inspectors, watchmen, conductors, and security guards

### 3.2 Field monitoring

#### 3.2.1 Monitoring devices

**Figure 2:**
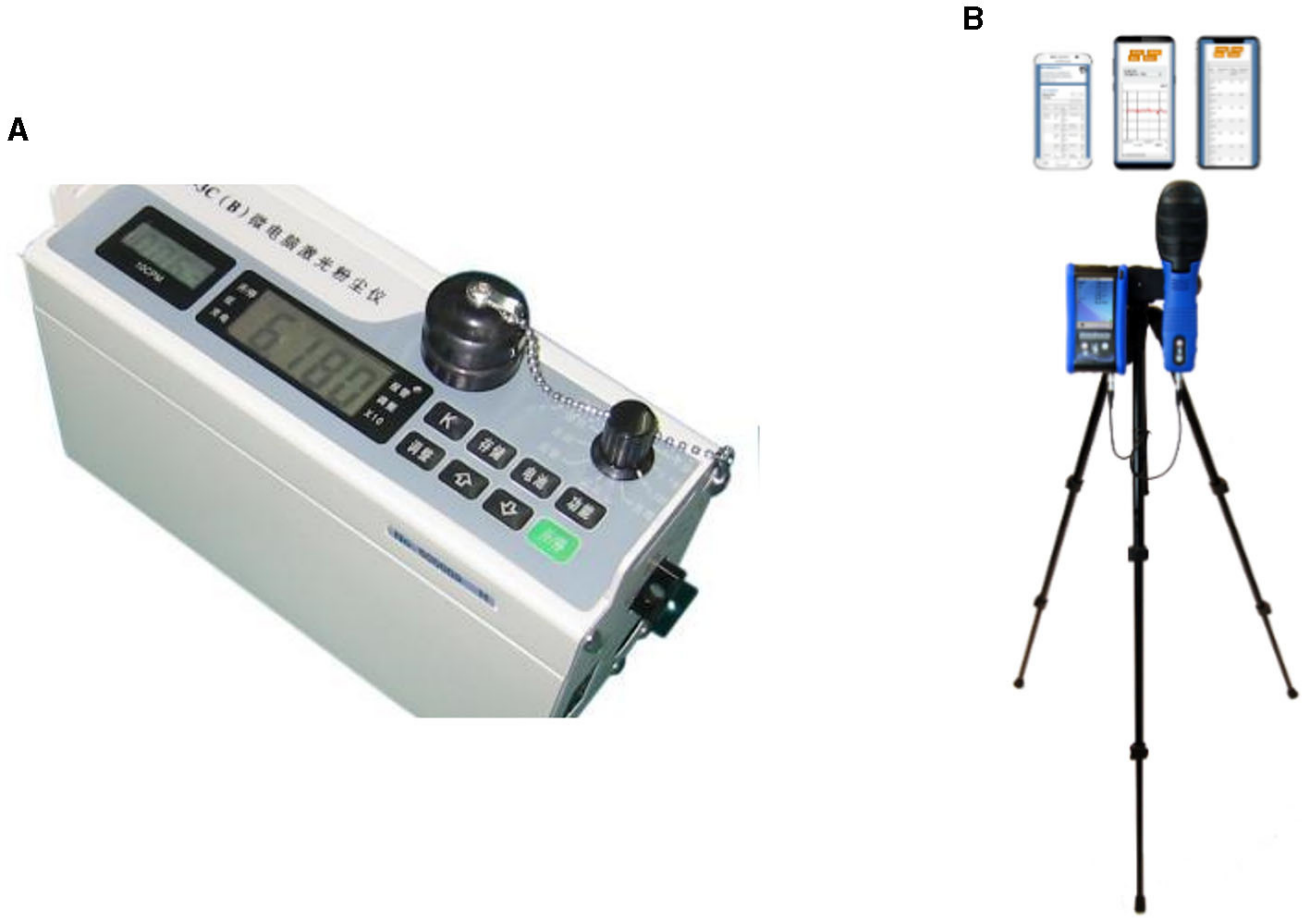
The adopted monitoring devices. **(A)** Laser dust meter LD-3C (B); **(B)** Multifunctional portable environmental quality inspection system instrument.

To reduce measurement errors, this study employed specialized instruments for monitoring and the operation and field monitoring of the instruments complied with standard requirements (47, 49). The mass concentration of PM10 was measured in real time using a laser dust meter LD-3C (B) (sensitivity = 0.001 mg/m^3^; precision = 10%) produced by Beijing Greenwood Innovation Digital Technology Co., Ltd. A multifunctional portable environmental quality inspection system instrument from Gray Wolf Sensing Solutions, LLC, Shelton, CT, USA (sensitivity = 0.01 mg/m^3^; precision = 10%) was used to monitor BTEX. The devices underwent routine verification and calibration by the Shanghai Institute of Measurement and Testing Technology, and the obtained results met the specified criteria for qualification. Additionally, prior to each measurement, zero calibration was diligently performed in accordance with the instrument instructions. The instrument readings are presented in a direct numerical display format. The adopted monitoring devices are shown in Figure 2.

#### 3.2.2 Sampling points

**Figure 3:**
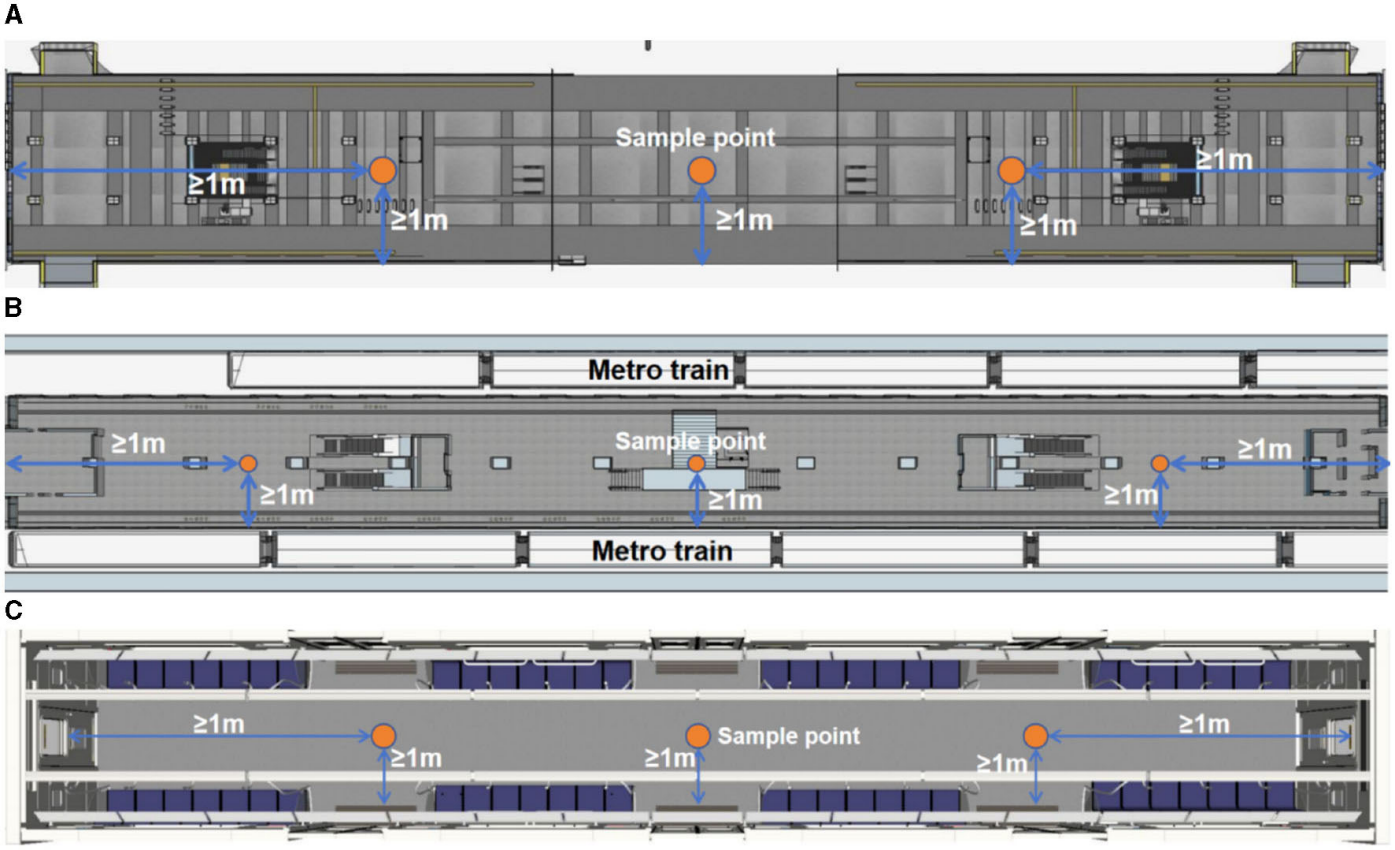
Distributions of sampling points in **(A)** station halls, **(B)** platforms, and **(C)** metro cabins.

Three monitoring sites were selected: station halls, platforms, and metro cabins. At least three sampling points were required for each monitoring site, and the distribution of the sampling points complied strictly with the latest national norms and standards (46–49), as shown in Figure 3. Each sampling point was monitored several times and the monitoring values we reported were the average level of the three measuring points in one area. A continuous sampling height in the breathing zone is adopted, specifically ranging from 0.5 to 1.5 m above the ground. The sampling point was located far from the strong-wind area (such as the side of air vents and screen doors) and at least 1 m from the wall and cabin. The sampling times were carefully scheduled to avoid rainy days and daily peak times. The sampling interval was 6 s. Besides, the instrument was carefully adjusted in the laboratory and underwent strict zero calibration before each measurement in the field. The monitoring was performed on the same day to avoid potential environmental changes. All laboratory operations and field monitoring were performed by trained laboratory assistants to avoid bias. This meticulous approach ensured that the monitoring process adheres to established standards, allowing accurate and reliable data collection.

#### 3.2.3 Data processing

After monitoring, concentration data were processed using SPSS version 22.0 (IBM, Armonk, NY, USA) and then presented in the form of “mean ± standard deviation (SD)” with individual 95% confidence interval. Some well-established standards for indoor air quality were utilized as reference, including the Hygienic Standards for Public Transport Equipment Waiting Rooms (GB9672-1996), Design Codes (GB50157-2013), and Code for Indoor Environmental Pollution Control of Civil Engineering (GB50325-2010). The limit concentration levels of PM10 and BTEX were identified as 0.25 and 0.5 mg·m^−3^, respectively.

### 3.3 Health damage assessment

#### 3.3.1 Exposure dose calculation

The exposure parameter method is a well-established method for calculating exposure doses proposed by the U.S. Environmental Protection Agency and is used to convert the monitored pollutant concentrations in Section 3.2 into the average daily exposure to pollutants per unit body weight of a metro staff or commuter. The primary calculation parameters include pollutant concentration, human physical characteristics, and exposure duration. Physical characteristics, such as breathing rate and body weight, are divided by age group. The discrepancy of pollutant concentration and time consumed in three areas are considered. The calculation formulas for the average daily doses of commuters and staff (ADD_C_ and ADD_W_, respectively) are shown in Equation (1) (50) and Equation (2) (42).


(1) $$\begin{array}{l} ADD_{C}=\frac{C\cdot IR\cdot ED\cdot EF\cdot EL}{BW\cdot AL} \end{array}$$

(2) $$\begin{array}{l} ADD_{W}=\overset{3}{\underset{i=1}{\sum }}\frac{IR\cdot C\cdot N_{i}}{BW} \end{array}$$

Where C is the pollutant concentration, mg/m^3^; IR is the respiration rate of staff or commuters, the amount of pollutants inhaled per unit time, m^3^/h; AL is the average time of exposure, d; ED refers to the long-run period of time during which a specific pollutant concentration persists within the environment, a; EF is the number of days the staff is exposed to the pollutants per year, d/a; EL is the time that the staff is exposed to pollutants per day, h/d; BW is the weight of staff or commuters, kg; Ni is the time commuters are exposed to site i per day, min/d; i = 1, 2, and 3 represent halls, platforms and metro cabins, respectively.

For the calculation, IR and BW indicator data were derived from the Manual of Chinese Exposure Parameters (51). In order to enhance the accuracy of calculations, human physical data sampled from the population in Jiangsu Province is utilized. The population is categorized based on age groups, and due to the significant age-related variations in children's body parameters, they have been further divided into more specific age ranges (50). The values of these parameters are listed in Table 2. The population's exposure per unit year is considered in the calculation; therefore, the ED value equals 1a, and the value of AL is 365d. The values of EF, EL, and Ni can be obtained through surveys and interviews based on the actual situation.

**Table 2:** Parameters for calculating the dose of exposure to the population.

	**Age**	**IR (m^3^/h)**	**BW (kg)**
Staff	/	1.29	62
Commuters	6–9	8.3	26.7
	9–12	9.9	38.4
	12–15	11.4	51.3
	15–18	12.3	57.4
	18–44	8.7	61.9
	45–59	8.7	63.5
	Above 60	7.2	60.3

#### 3.3.2 Health risk assessment

The calculated exposure doses were then converted into human health exposure risks. Health risks quantify the possibility of causing disease, disability, and health loss at a certain exposure dose. Equation (3) is used to assess carcinogenic risks, and Equation (4) analyzes the non-carcinogenic risk of pollutants (50). Notably, BTEX is a mixture of many chemical components, and only the major components (benzene, toluene, ethylbenzene, and xylene) are considered in most health-related studies (52–54). Among the four major pollutant types, only benzene is a direct carcinogen, whereas the other three types are indirect. The health damage caused by benzene is significant (55) and the values of the ISF and RFD indicators have been more thoroughly investigated than those of other compositions (56). Given its importance and data availability, this study quantifies the health risks and damage caused by benzene in the following sections. Referring to literature, the RFD values of PM10 and benzene in the metro system were 0.7 mg/(kg·d) (12) and 5 mg/(kg·d), respectively. The ISF values for PM10 and benzene were 0 and 0.0273 (kg·d)/mg, respectively (57).


(3) $$\begin{array}{l} R_{C}=ADD\;\cdot \;ISF \end{array}$$

(4) $$\begin{array}{l} R_{NC}=\frac{ADD}{RFD}\cdot 10^{-6} \end{array}$$

Where *R*_*C*_ represents the carcinogenic human health exposure risk (no dimension); ADD represents the average daily exposure of commuters or staff [ADDC or ADDW, mg/(kg·d)]; ISF represents inhalation slope factor of carcinogenic pollutants, (kg·d)/mg; *R*_*NC*_ represents the non-carcinogenic human health exposure risk (no dimension); and RFD is the reference dose of pollutants, mg/(kg·d).

#### 3.3.3 Health damage analysis

After health risk assessment, disability-adjusted life years (DALY) was adopted to quantify the related health damage. The DALY is a comprehensive metric used to quantify the burden of diseases by measuring the number of years lost due to disability and premature death. It presents the gap between the current health status of the population and the ideal health state of no one suffering from disease, disability, or longevity. The gap between chronological age and life expectancy is an important influencing factor, reflecting the duration of potential health effects. The types of health damage are determined by pollutants. Equation (5) is used to conduct the calculation (58, 59), and the related data values are summarized in Table 3 (60).


(5) $$\begin{array}{l} \text{DALY}=\text{n }\cdot \;\underset{\text{i}}{\sum }\text{R }\cdot \;{\text{Q}}_{\text{i}}\;\cdot \;{\text{W}}_{\text{i}}\;\cdot \;{\text{L}}_{\text{i}}\;\cdot \text{ P} \end{array}$$

where n is the number of exposure days, specifically, the total working days of staff or the number of days commuters took the metro, d; and R is the carcinogenic or non-carcinogenic health exposure risk (*R*_*C*_ or *R*_*NC*_). Qi represents the risk factor associated with disease type i and is quantified as the ratio of the risk in different damage types (no dimension). Wi denotes the impact factor of disease type i, which ranges from 0 to 1 (no dimension). Li signifies the impairment factor of disease type i, a, and P indicates the number of individuals affected by damage.

**Table 3:** Data values for health damage related parameters.

**Types of health damage**	**Q**	**W**	**L (a)**	**L (a)**							
			**Staff**	**Commuter**							
					**6–9**	**10–12**	**13–15**	**15–17**	**18–44**	**45–59**	**Above 60**
Death	0.13	1.00	L_0_	48.63	69.13	65.63	62.63	60.63	45.63	24.63	6.63
COPD	0.16	0.15	10	10	10	10	10	10	10	10	10
CVD	0.16	0.24	L_0_-5	43.63	64.13	60.13	57.63	55.63	40.63	19.63	1.63
Cerebrovascular disease	0.20	0.20	L_0_-5	43.63	64.13	60.13	57.63	55.63	40.63	19.63	1.63
Acute respiratory infection	0.35	0.08	0.04	0.04	0.04	0.04	0.04	0.04	0.04	0.04	0.04
Leukemia	1	0.75	L_0_	48.63	69.13	65.63	62.63	60.63	45.63	24.63	6.63

L_0_ refers to standard life expectancy, which is the difference between the average life expectancy and average age of the target population. The required life data were obtained from Son et al. (69).

#### 3.3.4 Weighting evaluation

Willingness-to-Pay (WTP) is a widely used approach for determining the highest price that consumers are willing to spend on a product, service, or a specific feature (61). In disaster economic analysis, the value of statistical life year (VSLY) refers to an individual's willingness to pay to reduce the risk of death per unit. Therefore, the total value of personal health damage can be obtained through the VSLY and DALY. In this study, the final health damage value was calculated based on the WTP to present the monetized value of health damage when someone suffers. Equation (6) calculates and quantifies the money value of health damage and provides a reference for health subsidy policies. The VSLY indicator is calculated using the value of statistical life (VSY) and utility discount rate (62), as shown in Equation (7).


(6) $$\begin{array}{l} \text{Health value}=\text{DALY }\cdot \text{ VSLY} \end{array}$$

(7) $$\begin{array}{l} \text{VSLY}=\frac{VSL}{\left[1-{\left(1+r\right)}^{-t}\right]/r} \end{array}$$

Where VSLY is the unit value of health risk, yuan/a; VSL is the value of statistical life, yuan; t is life expectancy, a; r is the utility discount rate, and its value is 5% (64).

## 4 Case application

### 4.1 Basic information

**Figure 4:**
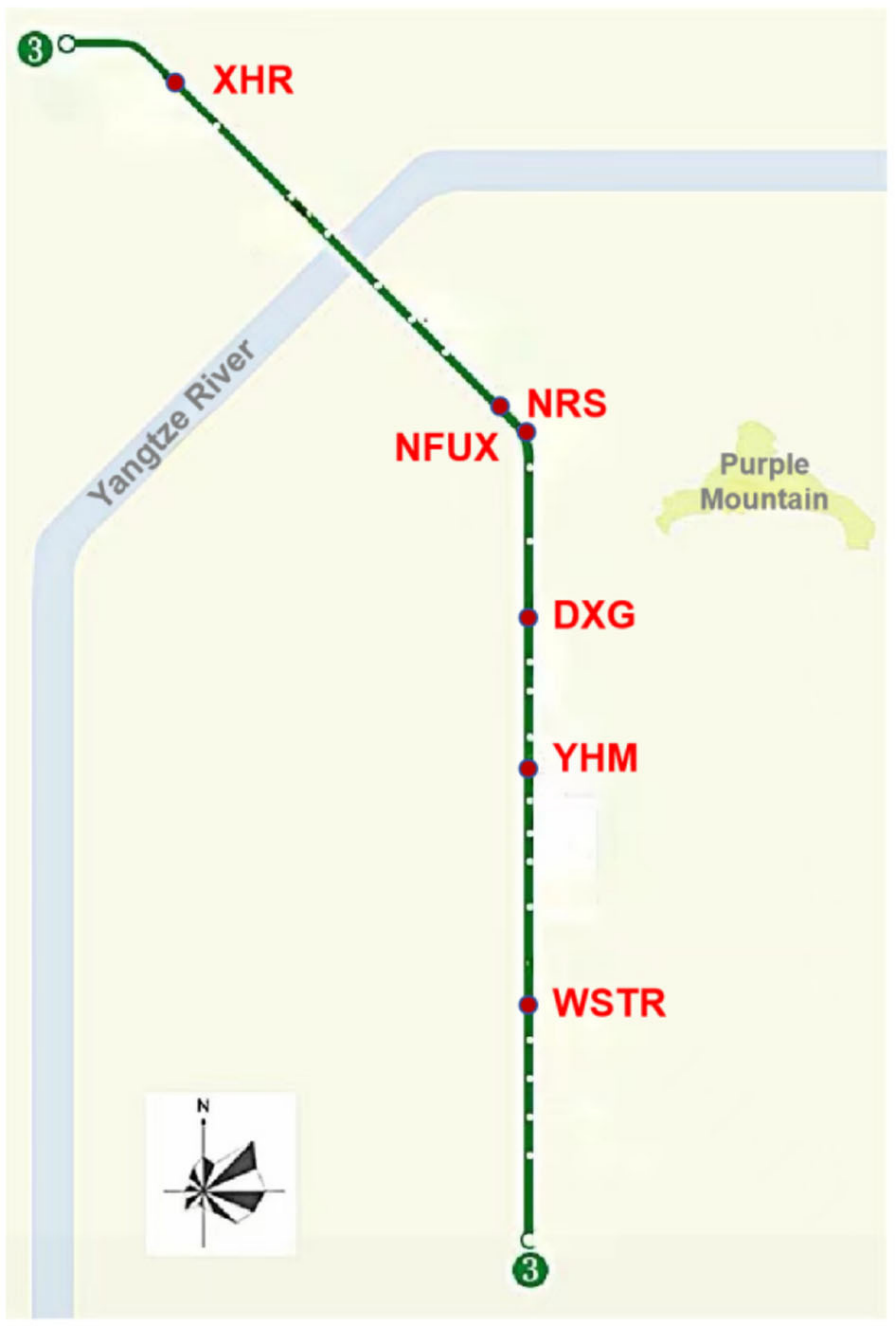
Map of Nanjing Metro Line 3.

In this study, Nanjing Metro Line 3 was used as a case study for the health damage assessment model. Nanjing Metro Line 3 officially opened in 2015, passing through the districts of Pukou, Gulou, Xuanwu, and Qinhuai in Nanjing, with a total length of 44.9 km. The route map of Metro Line 3, shown in Figure 4, has 29 stations, of which 28 are underground stations. This line connects major residential areas, the main business district, and Jiangning University Town, resulting in large passenger flow. Commuters have a wide age distribution and rich individual characteristics.

Compared with aboveground stations, underground stations are more airtight and conducive to the accumulation of pollutants. Therefore, six underground metro stations on Nanjing Metro Line 3 were selected to conduct field monitoring of air pollutants, as summarized in Table 4. They are all located in typical city center areas and suburban regions to ensure the representativeness and applicability of the monitoring results to a broader urban context. The selected metro stations cover typical categories, including suburban, interchange, and downtown. The halls and platforms of the selected stations were monitored. In addition, a random sampling method was used to select five trains for monitoring, with official codes 033034, 049050, 065066, 077078, and 083084.

**Table 4:** Information of the selected six monitor stations.

**Station**	**Category**	**Form**
Xinghuo Road (XHR)	Suburban station	Underground two-story island
Nanjing Railway Station (NRS)	Interchange station	Underground two-story twin-island
Nanjing Forestry University Xinzhuang (NFUX)	Downtown station	Underground two-story island
Da Xing Gong (DXG)	Interchange station	Underground three-story island
Yu Hua Men (YHM)	Downtown station	Underground two-story island
West of Shengtai Road (WSTR)	Suburban station	Underground two-story island

Additionally, the exposure parameters of Nanjing Metro crowds were obtained through field surveys. The daily working hours of Nanjing Metro staff are 12 h, and the working system is a 2-day holiday; therefore, the value of the EF indicator is 302 days. The average daily commuting time using the metro in Nanjing is 37 min; therefore, the average total daily commute time was set to 74 min (63). In particular, commuters stayed in three areas (station halls, platforms, and metro cabins) for 4, 6, and 64 min, respectively.

### 4.2 Field sampling

**Figure 5:**
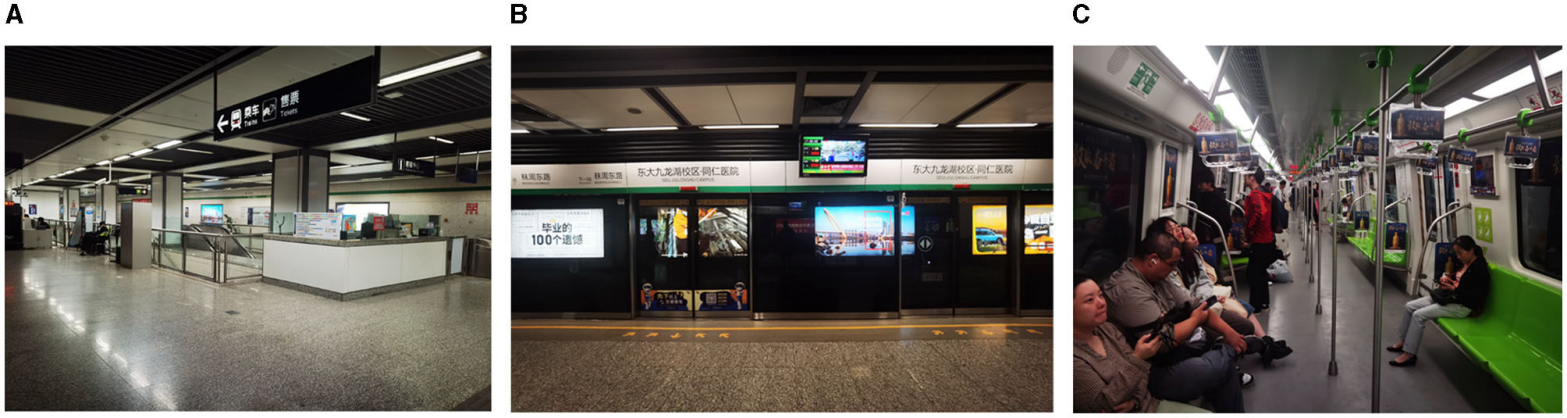
Different monitoring areas in the metro system. **(A)** Station hall; **(B)** Platform; **(C)** Metro cabin.

Field monitoring of pollutant concentrations was conducted in station halls and platforms of six selected metro stations and five metro metro cabins using the sampling devices described in Section 3. The monitoring areas for Metro Line 3 are shown in Figure 5. Each monitoring area had three sampling points and the sampling frequency of each monitoring point was 6 s. Therefore, the pollutant concentration in each monitoring area was the average value of all monitoring data in the area, as summarized in Supplementary Table 1. The average PM10 concentration levels in the platforms, station halls, and metro cabins are 0.185 ± 0.128 mg·m^−3^, 0.174 ± 0.135 mg·m^−3^, and 0.05 ± 0.017 mg·m^−3^, respectively. These data are 0.10 ± 0.01 mg·m^−3^, 0.10 ± 0.01 mg·m^−3^, and 0.10 ± 0.02 mg·m^−3^, respectively in the case of BTEX.

### 4.3 Damage assessment

With the monitored pollutant concentrations, health damages were assessed using the formulas and parameters described in Section 3.3. The calculation utilized the average pollutant concentration levels. It is important to mention that about the calculation of VSL, the per capita life value of the United States is $7, inflation rate of the dollar from January 2000 to June 2022 is 75.54%, per capita GDP of the United States in 2021 is 69,287.5 dollars, per capita GDP of Nanjing in 2021 is 27,051 dollars, and exchange rate between the US dollar and the yuan is 6.74. Therefore, the average VSL value for Nanjing is 4.797 × 10^6^ *yuan* (64). Some computational data for Equations (3–6) are summarized in Supplementary Table 2.

### 4.4 Results analysis

#### 4.4.1 Health damages at different metro stations

**Figure 6:**
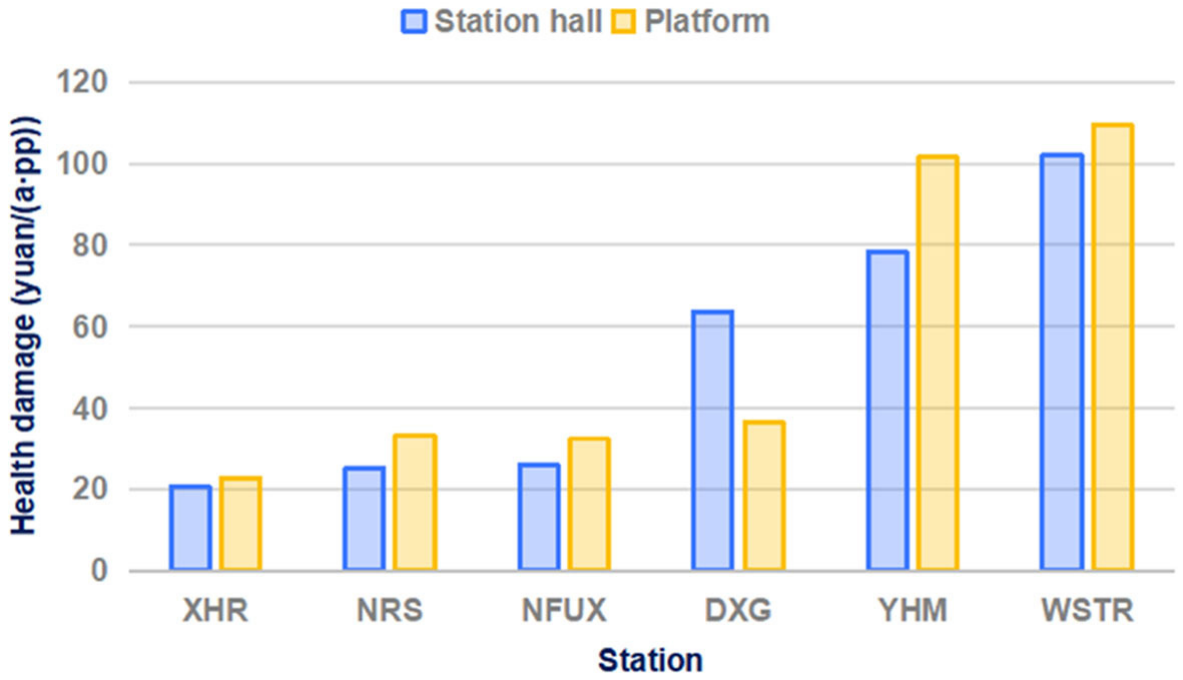
The health damage levels at different stations.

The health damage levels in the station halls and platforms of the six stations are compared in Figure 6. The health damage levels at the West of Shengtai Road (WSTR) station weare the highest at 0.338 yuan and 0.362 yuan in the hall and platform, respectively. These levels were nearly five times of the lowest levels at the Xinghuo Road (XHR) station (0.075 yuan in the hall and 0.068 yuan in the platform). The XHR station has the least damage because it is located in the suburbs with few people and is very close to the terminal of Line 3. Among the six stations, the health damage at the Yu Hua Men (YHM) and WSTR stations are relatively large, which may be because their entrances are close to intersections and vehicle exhaust emissions aggravate the poor air quality inside the stations.

At most stations, the health damage in station halls was much lower than that in the corresponding platforms. This discrepancy may arise from the platform environment being more favorable for pollutant accumulation, coupled with its proximity to the metro tracks. Station Da Xing Gong (DXG) was the only exception. This is a transit station located in the center of Nanjing. It has a very large flow of passengers on lines 2 and 3 entering and exiting through the hall, resulting in poor air quality.

#### 4.4.2 Health damage analysis of staff

**Figure 7:**
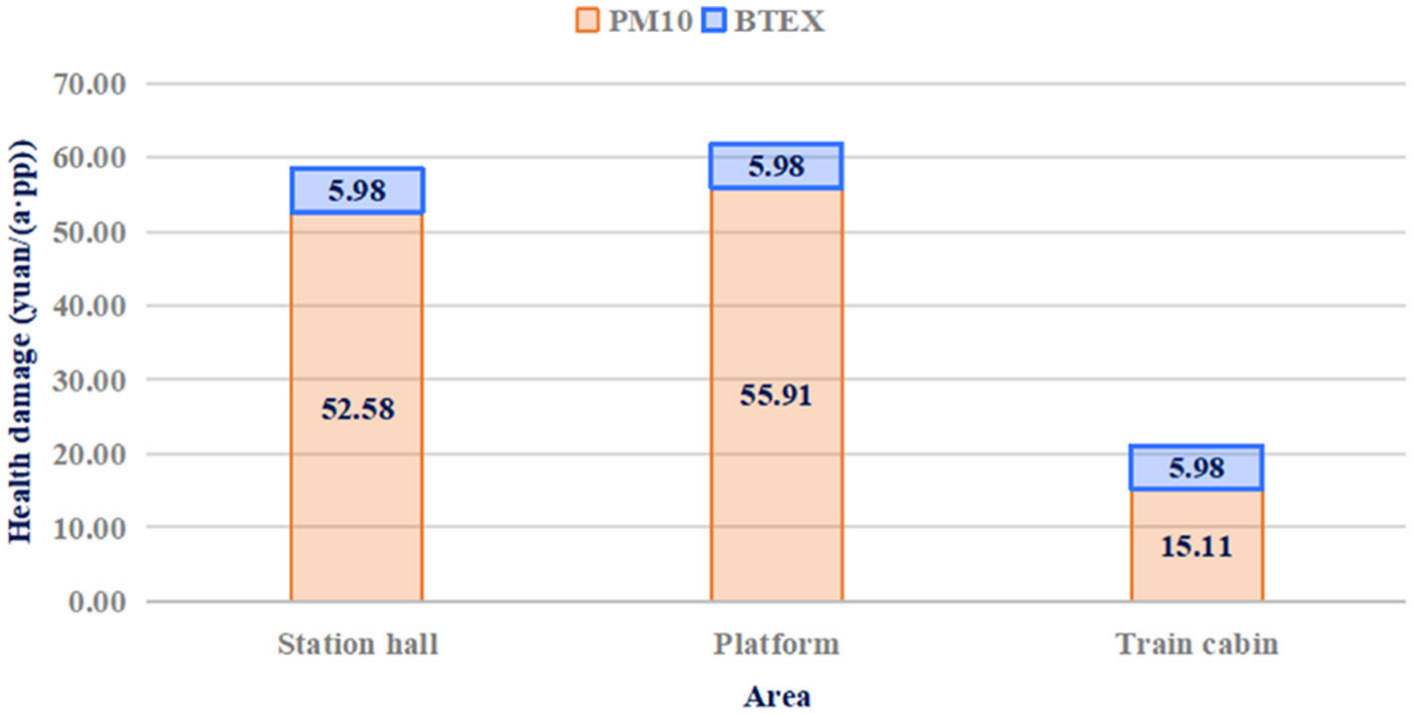
Health damage levels in different areas.

The pollutant concentrations were monitored at three different sites (hall, platform, and cabin) in the metro system, and the health damage to the staff was assessed, as shown in Figure 7. The health damage caused by PM10 in the hall, platform, and cabin was 52.58, 55.91, and 15.11 yuan, respectively. Health damages due to BTEX in the hall, platform and cabin are all 5.98 yuan. The health damage levels of BTEX in different areas were not significantly different; in contrast, the health damage caused by PM10 was greatly affected by the area.

The damage level in the cabin accounted for only 27.02% of the damage level in the other two areas. This may be because the cabin is in motion most of the time, which is not conducive to pollutant accumulation. Staff in station halls and platforms (station attendants, safety officers, and security inspectors) suffer more serious health damage than those in metro cabins (drivers and driving attendants). Metro companies should take effective measures to protect their staff, particularly those working at stations and concourses.

#### 4.4.3 Health damage analysis of commuters

**Figure 8:**
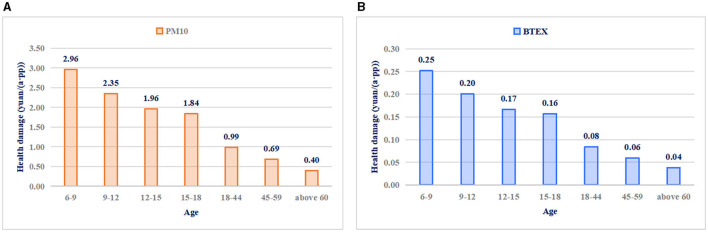
Health damage due to **(A)** PM10 and **(B)** BTEX of commuters at different ages.

Figure 8 shows the health damage levels of commuters of different ages; significant differences can be observed. Health damage values represent the cumulative damage experienced by commuters in station halls, platforms, and metro cabins. The health damage values due to PM10 to commuters in the seven age groups were 2.96, 2.35, 1.96, 1.84, 0.99, 0.69, and 0.40 yuan, respectively, whereas those due to BTEX were 0.25, 0.20, 0.17, 0.16, 0.08, 0.06, and 0.04 yuan, respectively. In the same polluted environment, the health damage suffered by commuters of different ages may differ significantly. This phenomenon can be explained by the fact that younger commuters weigh less but have higher breathing rates.

**Figure 9:**
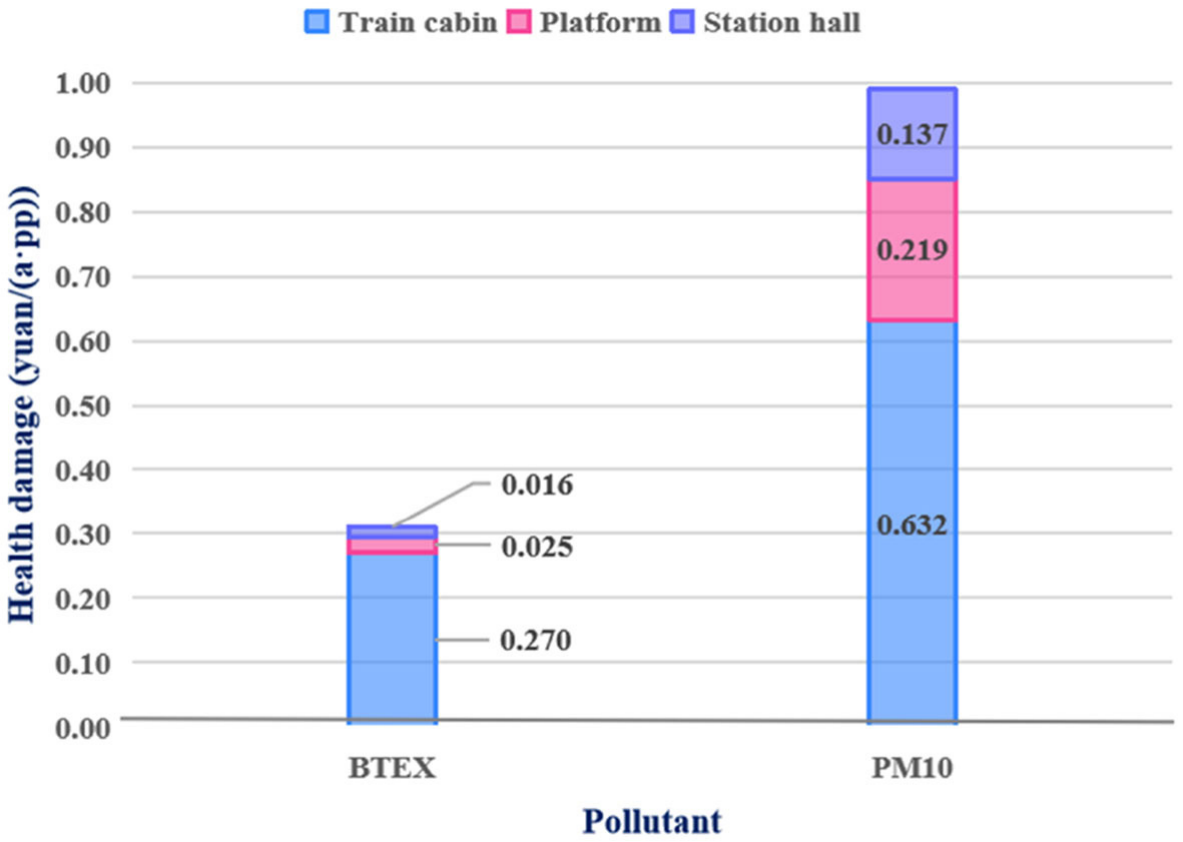
Health damage distribution of commuters at 18–44 years old.

The 18–44 years old group was the main group in the metro; therefore, this group was selected for a more detailed analysis, as shown in Figure 9. For a commuter, the health damages due to PM10 and BTEX are 0.99 yuan and 0.31 yuan each day, respectively. Most health damage occurred in the cabin, at 64% (0.632 yuan) and 87% (0.270 yuan) in the cases of PM10 and BTEX, respectively. The exposure time of commuters in the metro cabins was much longer than that in the other two places. Therefore, commuters are advised to improve their self-protection awareness and wear protective masks. The health damage suffered by the platforms ranked second. Many metal particles are generated by the friction between the brake pad and track when the train pulls in and out of the station.

## 5 Discussion

### 5.1 Monitoring time selection

In this study, pollutant monitoring at each station was conducted during the ventilation season. The sampling time was carefully selected, and monitoring was completed on the same day. Previous studies have demonstrated that crowd density can affect monitoring. In a study at Philadelphia metro stations (65), pollutant concentrations were compared at various times of the day, revealing higher levels during peak crowding hours. Aarnio et al. (66) also highlighted the effect of crowd density on pollutant concentrations. Passi et al. (28) found that the movement of a significant number of passengers and their respiration can lead to the re-suspension of settled particulate matter, causing more severe harm to the human body. Poor ventilation is considered the main cause of pollutant accumulation during peak periods of human traffic.

Meteorological condition is another influencing factor. Martins et al. (17) and Roy (11) both found that pollutant levels are higher during the cold season than during the warm season. Ventilation differences between seasons was deemed to the main reason (28). Additionally, the operation of fuel heating systems in winter may also have contributed to the rise in pollutant concentrations. Seasonal factors may also affect the exposure time, transportation frequency and breathing rate of the population. Hence, there are also seasonal differences in health damage.

This study carefully selected monitoring times and considered the possible influence of crowd density. It aims to provide more accurate and reliable data within a metro system during typical ventilation periods. A future perspective is to consider more diversified period divisions and monitoring scenarios, which will be conducive to comprehensively considering factors such as crowd density and seasons and improving the validity.

### 5.2 Pollutant control measures

The application in this study indicates that the health damage to commuters and staff cannot be ignored. Therefore, it is imperative to implement relevant pollutant mitigation measures. Optimizing brake pad materials and using reasonable braking methods such as changing the braking mechanism can reduce pollutant emissions (67). Furthermore, the installation of platform screen doors, ventilation measures, and filtration devices can effectively mitigate pollutant accumulation. The installation of platform screen doors has been shown to reduce 16–30% PM10 concentration (18, 68). Integrating magnetic filters into ventilation systems can effectively eliminate up to 50% of PM10 (69). Additionally, accelerating the exchange of outdoor fresh air with indoor air in metro stations and reducing the recirculation of indoor polluted air are important measures for controlling pollutants (27). Tu et al. (20) found that a well-designed ventilation and air exchange system can effectively reduce particle concentrations on subway platforms.

To avoid interference from different pollutant control measures, the same metro line was selected in this study for field monitoring. The braking devices and ventilation equipment were identical, and the six selected metro stations were equipped with screen doors on the platform. For future research, it would be worthwhile to compare the differences in health damage caused by the abovementioned control measures, and for a specific metro system, comprehensively considering the cost and pollution control effect, choose the appropriate equipment and control measures.

## 6 Conclusion

This study developed a health damage assessment model for commuters and staff in metro systems based on field monitoring, consisting of three modules (goal and scope definition, field monitoring, and health damage assessment). The model considered two main air pollutants, PM10 and BTEX, and selected three monitoring sites (station halls, platforms, and metro cabins) based on the spatial distribution of pollutants. The established model was applied to Line 3 of the Nanjing Metro, and the findings are as follows:

1) Health damage at different types of stations was compared, and the results showed that the health damage from pollutants in different metro stations varied greatly and was affected by factors such as station type, location, and distribution of entrances and exits. In addition, the study revealed that the health damage to the platform is usually higher than that to the hall, but the opposite is true at the transfer station, which is a valuable discovery for the design of the interior space of the metro station.
2) Regarding the health assessment of the metro staff, the health damage suffered by staff in station halls and platforms was much more serious than that suffered by staff in metro cabins. Considering the health damage to staff caused by the two types of pollutants, PM10 contributed the most, demonstrating the importance of focusing on staff health.
3) The damage suffered by commuters was analyzed from multiple perspectives, such as age, pollutants, and exposure sites. In the health damage assessment of commuters, young people, especially children, suffered significantly more health damage than commuters of other ages. The damage caused by pollutants to commuters gradually decreased with increasing age. Additionally, for the main commuter population, the study found that the damage caused by BTEX and PM10 to people mainly occurred in metro cabins, while the health damage caused by PM10 in station halls and platforms cannot be ignored, which provides an important consideration for the research and protection of health damage of commuters.

The study provides the following contributions: (1) Analyzed the health impact of the metro system on commuters and staff and quantifies related damage. (2) Obtained pollutant data through field monitoring thereby presenting a realistic scenario for assessment models. (3) Identified areas with significant health impacts, offering a reference for improving air quality and strengthening control measures within the Nanjing Metro system. (4) Monetized health damage to assist metro companies identifying appropriate occupational health subsidies and support policies.

However, this study still can be improved. Special groups, such as the disabled, were not studied, and future research can pay more attention on this group. Besides, some detailed factors were not fully considered in this paper, such as the seasonal factors, walking speed and crowd density. Future research could develop more comprehensive monitoring plans and incorporate more up-to-date disease reports.

## Data availability statement

The original contributions presented in the study are included in the article/Supplementary material, further inquiries can be directed to the corresponding author.

## Ethics statement

All our data collection and filed experiments have obtained the approval and support from the Nanjing Municipal Center for Disease Control and Prevention and the Nanjing Metro Operating Company.

## Author contributions

SS: Writing – original draft, Funding acquisition, Investigation, Methodology, Supervision. SL: Writing – original draft, Methodology, Data curation, Visualization, Writing – review & editing. YD: Supervision, Writing – review & editing, Visualization. PM: Writing – review & editing, Data curation, Investigation, Supervision, Resources. DC: Writing – review & editing, Supervision, Formal analysis.
