# Supplementary material for: Health damage assessment of commuters and staff in the metro system based on field monitoring—A case study of Nanjing
Source: Front Public Health. 2024 Jan 11;11:1305829. doi: 10.3389/fpubh.2023.1305829 (PMC10808693; doi:10.3389/fpubh.2023.1305829)
Supplement: Supplementary file 1 [file Table_1.DOCX]

Supplementary Material

Health damage assessment of commuters and staff in the metro system based on field monitoring – A case study of Nanjing

Shu Su^1^, Shuhao Li^1^, Yujie Ding^1^, Peng Mao^2*^, Dan Chong^3^

^1^Department of Construction and Real Estate, School of Civil Engineering, Southeast University, Nanjing 211189, China

^2^Department of Engineering Management, School of Civil Engineering, Nanjing Forestry University, Nanjing 210037, China

^3^Department of Management Science and Engineering, School of Management, Shanghai University, 200444, China

*** Correspondence:**Peng Mao
maopeng@njfu.edu.cn

# Supplementary Tables

**Table S1.** The monitored pollutant concentrations at six stations on Nanjing Metro Line 3.

| Station | Monitoring site | PM10 (mg·m-3) | BTEX (mg·m-3) |
| --- | --- | --- | --- |
| XHR | Station hall | 0.068±0.007 | 0.09±0.000 |
|  | Platform | 0.075±0.009 | 0.09±0.000 |
| NRS | Station hall | 0.083±0.008 | 0.10±0.000 |
|  | Platform | 0.110±0.024 | 0.11±0.000 |
| NFUX | Station hall | 0.086±0.010 | 0.11±0.000 |
|  | Platform | 0.107±0.033 | 0.11±0.000 |
| DXG | Station hall | 0.211±0.113 | 0.10±0.000 |
|  | Platform | 0.121±0.008 | 0.11±0.000 |
| YHM | Station hall | 0.259±0.023 | 0.09±0.000 |
|  | Platform | 0.336±0.035 | 0.09±0.000 |
| WSTR | Station hall | 0.338±0.198 | 0.09±0.000 |
|  | Platform | 0.362±0.113 | 0.10±0.000 |
| / | Train cabin | 0.050±0.017 | 0.10±0.000 |
