# Supplementary material for: Health damage assessment of commuters and staff in the metro system based on field monitoring—A case study of Nanjing
Source: Front Public Health. 2024 Jan 11;11:1305829. doi: 10.3389/fpubh.2023.1305829 (PMC10808693; doi:10.3389/fpubh.2023.1305829)
Supplement: Supplementary file 2 [file Table_2.DOCX]

Supplementary Material

Health damage assessment of commuters and staff in the metro system based on field monitoring – A case study of Nanjing

Shu Su^1^, Shuhao Li^1^, Yujie Ding^1^, Peng Mao^2*^, Dan Chong^3^

^1^Department of Construction and Real Estate, School of Civil Engineering, Southeast University, Nanjing 211189, China

^2^Department of Engineering Management, School of Civil Engineering, Nanjing Forestry University, Nanjing 210037, China

^3^Department of Management Science and Engineering, School of Management, Shanghai University, 200444, China

*** Correspondence:**Peng Mao
maopeng@njfu.edu.cn

# Supplementary Tables

**Table S2.** Health damage calculation data.

|  | Area | Age | R(10^-8^) | | DALY (10^-7^ a) | | Health damage  (yuan/(a·pp)) | |
| --- | --- | --- | --- | --- | --- | --- | --- | --- |
|  |  |  | PM10 | benzene | PM10 | benzene | PM10 | benzene |
| Staff | Station hall | / | 6.206 | 0.5558 | 18712.21 | 2126.78 | 52.58 | 5.98 |
|  | Platform | / | 6.599 | 0.5558 | 19895.17 | 2126.78 | 55.91 | 5.98 |
|  | Train cabin | / | 1.783 | 0.5558 | 5377.07 | 2126.78 | 15.11 | 5.98 |
| Commuter | / | 6-9 | 0.222 | 0.0523 | 1156.76 | 337.73 | 2.96 | 0.86 |
|  | / | 9-12 | 0.184 | 0.0434 | 907.64 | 265.26 | 2.35 | 0.69 |
|  | / | 12-15 | 0.159 | 0.0374 | 748.35 | 218.56 | 1.96 | 0.57 |
|  | / | 15-18 | 0.153 | 0.0360 | 698.31 | 203.96 | 1.84 | 0.54 |
|  | / | 18-44 | 0.101 | 0.0236 | 343.33 | 100.40 | 0.99 | 0.29 |
|  | / | 45-59 | 0.098 | 0.0230 | 178.17 | 52.31 | 0.69 | 0.20 |
|  | / | 60-79 | 0.085 | 0.0201 | 38.36 | 11.56 | 0.40 | 0.12 |
